# What Do Single-Cell Models Already Know About Perturbations?

**DOI:** 10.3390/genes16121439

**Published:** 2025-12-02

**Authors:** Andreas Bjerregaard, Iñigo Prada-Luengo, Vivek Das, Anders Krogh

**Affiliations:** 1 Department of Computer Science, University of Copenhagen, 2100 Copenhagen, Denmark; 2 Center for Health Data Science, Department of Public Health, University of Copenhagen, 2100 Copenhagen, Denmark; inlu@di.ku.dk; 3 Center for Genomic Medicine, Rigshospitalet, Copenhagen University Hospital, 2100 Copenhagen, Denmark; 4 Integrated Omics, AI and Analytics, Development, Novo Nordisk A/S, 2860 Søborg, Denmark; vvda@novonordisk.com

**Keywords:** generative models, explainable AI, machine learning, gene expression, in silico perturbations, single-cell RNA sequencing, agentic AI

## Abstract

**Background**: Virtual cells are embedded in widely used single-cell generative models. Nonetheless, the models’ implicit knowledge of perturbations remains unclear. **Methods**: We train variational autoencoders on three gene expression datasets spanning genetic, chemical, and temporal perturbations, and infer perturbations by differentiating decoder outputs with respect to latent variables. This yields vector fields of infinitesimal change in gene expression. Furthermore, we probe a publicly released scVI decoder trained on the CELL×GENE Discover Census (∼5.7 M mouse cells) and score genes by the alignment between local gradients and an empirical healthy-to-disease axis, followed by a novel large language model-based evaluation of pathways. **Results**: Gradient flows recover known transitions in *Irf8* knockout microglia, cardiotoxin-treated muscle, and worm embryogenesis. In the pretrained Census model, gradients help identify pathways with stronger statistical support and higher type 2 diabetes relevance than an average expression baseline. **Conclusions**: Trained single-cell decoders already contain rich perturbation-relevant information that can be accessed by automatic differentiation, enabling in-silico perturbation simulations and principled ranking of genes along observed disease or treatment axes without bespoke architectures or perturbation labels.

## 1. Introduction

Modeling perturbation response in cells is essential for understanding gene function, regulatory effects, and drug response. Single-cell RNA sequencing (scRNA-seq) provides high-resolution snapshots of cellular states and captures implicit gene–gene interactions. Although conditional knockout (cKO) experiments coupled with scRNA-seq can help reveal gene function, these experiments are prone to biases [1] and are costly across multiple conditions. Consequently, computational approaches that simulate perturbations from existing single-cell data are attractive; these could offer a scalable alternative for systematic analyses.

Generative models are widely used to learn low-dimensional *latent* representations of single-cell data and to reconstruct gene expression from such representations [2,3]. Beyond reconstruction, these models may implicitly capture cell–state transitions and regulatory elements. Recent methods explicitly learn perturbation mappings with supervision or mechanistic priors, including scGen and trVAE [4,5], regulatory network-based approaches such as CellOracle [6], and optimal transport formulations [7]. Other works study generalization to unseen doses, combinations, or cell types [8,9]. A core open question is how much perturbation knowledge can be extracted from a trained generative model *without* bespoke supervision or architectures. We hypothesize that generative models already encode structure ready to be queried without any supervision.

We ask a simple question: *What do single-cell models already know about perturbations?* Rather than using any supervision, we take a step backwards to explore what generative models have already learned. The goal is a simple, model-agnostic procedure that requires only a decoder, generalizes to arbitrary outputs (genes, treatments, cell age), and yields vector fields—perturbation flow maps—that aid interpretation and hypothesis generation.

**Figure 1:**
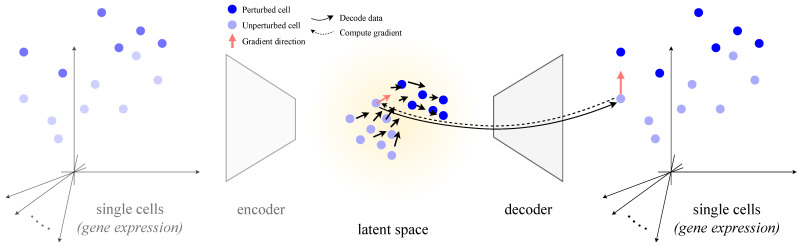
Methodological overview: A trained decoder maps single-cell representations from a latent space to a gene expression space. *Latent space* denotes the possible cell representation inputs for the decoder. We systematically compute gradients of an output gene with respect to the latent variables to show how a gene (or other perturbation) acts on the latent space. Because gradients are taken only through the decoder, any cell encoder is unnecessary and can safely be ignored.

Using trained single-cell decoders, we read out such perturbation fields by simply differentiating outputs with respect to latent variables (refer to Figure 1). The method requires minimal implementation overhead and can be applied to a multitude of existing models due to its *post hoc* nature. In brief, decoders already encode a usable perturbation structure that can be queried without supervision or modifications. Our highlights are as follows:

- **A simple, model-agnostic gradient probe** that turns any single-cell decoder into a simulator of infinitesimal perturbations over its outputs—no need for labeled samples or tailored architectures.
- **Arbitrary perturbation types** can be added by training lightweight task heads.
- **Pretrained models at scale** reveal flows aligned with *type 2 diabetes mellitus* (T2D) when probing an scVI decoder, enabling hypothesis testing without task-specific training.
- **Evaluating gene set analyses with an LLM** (large language model) opens a new direction for understanding the quality of gene set enrichments.

Code is made available on https://github.com/yhsure/perturbations (accessed on 9 November 2025) along with data references and other materials.

## 2. Materials and Methods

### 2.1. Data

We first analyze three public scRNA-seq datasets: *Irf8*-cKO *Mus musculus* (*M. m.*) brain macrophages [10], cardiotoxin-induced *M. m.* muscle injury [11], and *Caenorhabditis elegans* (*C. e.*) embryogenesis [12]. This collection treats diverse perturbations; collectively they span genetic (transcription factor knockout), chemical (toxin exposure), and temporal (developmental time) perturbation types across tissues and species. Datasets are already processed in the original studies, which apply different software to arrive at count matrices.

The *Irf8*-cKO dataset aggregates microglia and choroid-plexus border-associated macrophages (CP-BAM) from wild type (WT) and conditional knockout (cKO) animals. Here, *Irf8* is particularly a regulator of *microglial* identity, yielding a strong signal for single-gene knockdown and co-regulation analyses.

The cardiotoxin dataset profiles skeletal muscle under control and toxin treatment, with additional factors for diet (normal vs. high-fat) and sampling time (days 0, 4, 7); we use the binary treatment indicator as the supervised target and ignore other covariates.

The *C. e.* dataset is a whole-embryo time course with embryo time annotations (minutes after first cleavage) spanning many cell lineages, enabling evaluation of continuous temporal gradients without any external interventions.

We trained one model per dataset (no cross-dataset integration). We start from the respective unique molecular identifier (UMI) counts—i.e., the initial, unnormalized, and unscaled counts as provided—and perform our own uniform filtering and preprocessing as described in Section 2.2. Datasets were each partitioned into 82% of cells for training, 9% for validation, and 9% for testing. A basic overview of the data appears in Table 1.

In addition, we use a pretrained model based on the CELL×GENE Discover Census (5.7 M mouse cells) [13,14] to study T2D in pancreatic islets. We select islet cells from the Census dataset and randomly subsample 10,000 cells for analysis. We use the released decoder and embeddings as is to compute gradient fields; no additional normalization is applied beyond the model’s own preprocessing.

### 2.2. Preprocessing and Filtering

Preprocessing and filtering steps were identical across datasets (excluding the pretrained model). Cells were retained if they had at least 200 non-zero genes, at least 500 total counts, and fewer than 5000 non-zero genes. Genes having non-zero counts in less than 5 cells were not included. Raw counts were scaled by the mean count per cell and log1p-transformed to select highly variable genes with Scanpy; the inverse transform was then applied to recover raw counts for model training. These counts are to be used for reconstruction targets when assuming a count distribution. For the *Irf8* set, highly variable gene (HVG) selection is adjusted to include *Irf8*. Our models are trained on the HVG subset.

**Table 1:** Overview of scRNA-seq datasets applied in this study. The last row of CELL × GENE denotes a subset of 10,000 cells (from ∼5.7 M total cells) which we analyze in greater depth; it is italicized as we do not use this data for training. Their pretrained model covers 8000 output genes. HVGs denote highly variable genes; *M. m.* denotes *Mus musculus*; *C. e.* denotes *Caenorhabditis elegans*.

Dataset	Cells	Transcripts	HVGs	Reference
*Irf8* knockout *M. m.* brains	13,931	14,581	3451	Van Hove et al. [10]
Cardiotoxin *M. m.* injury	53,230	21,809	1950	Takada et al. [11]
*C. e.* embryogenesis	85,951	17,711	1832	Packer et al. [12]
*CELL × GENE islet subset*	*10,000*	*8000*	*n/a*	*CZI Cell Science Program et al. [14]*

### 2.3. Base Model: Negative Binomial β-VAE

Our model-agnostic approach requires a *base model* before we can infer any perturbation effects. Such a model describes the mapping from latent representations to gene expression. Specifically, we train β-variational autoencoders (β-VAEs) [15] with a negative binomial (NB) output distribution to account for overdispersion observed in expression data [3,16,17]. Single-cell models commonly use this backbone [18]. The *decoder* module then acts as our base model. The NB for each output gene is parameterized as a mean *m* and dispersion *r*:

(1) $$\mathrm{NB}\left(k;m,r\right)=\frac{\Gamma (k+r)}{k!\;\Gamma (r)}{\left(\frac{m}{r+m}\right)}^{k}{\left(\frac{r}{r+m}\right)}^{r},$$

where *k* is the observed count for a gene. The decoder outputs *m* scaled by the mean count for the sample, and one *r* is learned as a free parameter for each gene (shared across cells). Training minimizes the β-VAE objective [15]:

$$L_{\beta }\left(x;\theta ,\phi \right)=\underset{\mathrm{NB}\;\mathrm{reconstruction}}{\underset{︸}{E_{q_{\phi }\left(z|x\right)}\;\left[-\log p_{\theta }\left(x|z\right)\right]}}+\underset{\mathrm{latent}\;\mathrm{regularization}}{\underset{︸}{\beta \;\mathrm{KL}\;\left(q_{\phi }\left(z|x\right)\;|\;p\left(z\right)\right)}},$$

where $x\in N^{n\_\mathrm{genes}}$ is a cell’s gene-count vector, $z\in R^{d}$ a latent variable, θ/ϕ the decoder/encoder parameters, p(z)=N(0,I) the prior, and $p_{\theta }\left(x|z\right)$ an NB with mean $m_{\theta }\left(z\right)$ and gene-wise dispersion *r*. Compared to the NB approach, VAEs with other likelihoods result in lower accuracy [3,19]. We implement our model in PyTorch v2.5 with early stopping and linearly anneal β to a small final value, resulting in almost an autoencoder. The encoder module used ReLU activations, two hidden linear layers with sizes 512 and 256, and a latent dimensionality of 32 or 2. The β-VAE had a mirrored decoder module and was trained on one dataset at a time with Adam [20]; refer to Appendix B. The NB distribution is convenient for modeling raw counts from the decoder but is non-trivial to design for the encoder, which in turn takes traditional mean-scaled and log1p-transformed counts. The encoder is not strictly necessary, as the decoder could be trained on its own [21,22]; however, it is practical for larger datasets.

Pretrained models from hubs like scvi-hub [23] are very similar to our setup. To show how these deposited models can be utilized as base models, we further download an scVI model trained on the CELL×GENE Discover Census [13] in order to analyze T2D in mice. We did not finetune this model. All results are directly computed from the deposited decoder by differentiating gene outputs with respect to its latent variables. Further details on pretrained scVI models are found in Appendix A.

### 2.4. Core Idea: Perturbations from Decoder Gradients

The generative decoder learns how directions in a latent space relate to gene expression. We simulate perturbations by following the gradient of gene expression from an initial latent representation. Specifically, for a latent sample $z_{t}$, the perturbed sample is given by

(2) $$z_{t+1}=z_{t}+\delta \nabla y_{i}\left(z_{t}\right)$$

where δ is the perturbation stepsize and $\nabla y_{i}\left(z\right)$ is the derivative of the *i*-th gene expression output with respect to *z* (the *gradient*). A negative δ thus simulates decreasing gene expression (knockdown), while a positive δ simulates overexpression. Rather than selecting a specific starting cell, gradients are sampled across the latent space. To de-clutter visualizations, we mask away regions distant from training samples.

Arbitrary perturbations can be analyzed similarly by introducing an auxiliary output variable and a matching loss term in a multi-task setup. For treatment analysis, this can be a categorical or continuous variable indicating treatment type or dosage. Existing models can be adapted either through finetuning or by adding a new linear layer. This was used to model cardiotoxin response and *C. e.* embryo development.

For higher-dimensional latent spaces, we compute gradients at locations subsampled from existing data. The volume of Euclidean space grows exponentially with its dimensionality, so uniformly covering the latent volume would require an infeasible number of samples. Dimensionality reduction is subsequently used to project samples and gradient vectors. Here, PCA allows projection of the perturbation gradients directly, and could be followed by interpolating onto a grid (conveniently implemented using scipy.interpolate.griddata). In UMAP, the projection is highly non-linear and requires the encoding of perturbed endpoints into a new list, which is concatenated to the data before calculating the UMAP. Afterwards, the list is used to reconstruct the perturbation vectors.

### 2.5. Scoring Genes by Their Alignment with a Healthy-to-Disease Axis

Gradient directions can be used to *score* and *rank* genes according to an observed perturbation. Our technique is shown clearly in Appendix C. For an experimental perturbation (e.g., healthy vs. disease), we define the latent perturbation axis *a* as the mean displacement between groups:

(3) $$a\;=\;{\bar{z}}_{\mathrm{perturbed}}-{\bar{z}}_{\mathrm{unperturbed}}$$

We score gene *i* by the average cosine similarity between its gradient field and the axis in Equation (3) over a set of evaluation points Z (observed cells):

(4) $$\begin{array}{cc} s_{i}\; & =\;{\mathrm{avg}}_{\;z\in Z}\;\cos\;\left(∠(\nabla_{z}y_{i}\left(z\right),\;a)\right) \end{array}$$

(5) $$\begin{array}{cc} \; & =\;\frac{1}{\left|Z\right|}\sum_{z\in Z}\frac{\nabla_{z}y_{i}{\left(z\right)}^{⊤}a}{{∥\nabla_{z}y_{i}\left(z\right)∥}_{2}\;{∥a∥}_{2}}\;. \end{array}$$

Here, $s_{i}\in \left[-1,1\right]$ quantifies a directional agreement: larger values indicate that gradients align with the healthy → disease transition. As we have a large degree of freedom in sampling Z, one may compute the score for, e.g., purely healthy or disease samples. The range of $s_{i}$ covers gradients pointing in the reverse direction ($s_{i}=-1$), orthogonally ($s_{i}=0$), or the same direction ($s_{i}=1$).

### 2.6. Evaluating Pathways for a Complex Disease

Upon gene enrichment, we select the top 200 genes with the largest absolute scores. These gene sets are analyzed with *WebGestalt* overrepresentation analysis [24], using pathways from WikiPathways [25]. Pathway results are next mechanistically interpreted using an LLM-in-the-loop pipeline. We run the following prompt for each pathway, using GPT-5 (OpenAI. 2025. Model *gpt-5-mini-2025-08-07*) with reasoning and web-search enabled:

**Prompt 1.** *You have an expert perspective in bioinformatics. Is* [pathway] *highly relevant for type 2 diabetes mellitus in Mus musculus? Answer with Yes or No. Afterwards, describe shortly your explanation for whether the pathway involves type 2 diabetes, providing references for your claims.*

We find pathway interpretations from this stage to already be highly accurate. To combat non-determinism, we re-run the same prompt thrice and feed answers into *Prompt 2:*

**Prompt 2.** *You have an expert perspective in bioinformatics. Your task is to very concisely judge whether a pathway is relevant for type 2 diabetes mellitus (T2D) in Mus musculus. When asked whether* [pathway] *is highly relevant for T2D in Mus musculus, these were your answers from three distinct runs:*

*Answer 1:* [answer 1]

*Answer 2:* [answer 2]

*Answer 3:* [answer 3]

*Now give your final critical verdict with a Yes or No, and describe very concisely your explanation (with a few sentences at most), using correct scientific references.*

Results are used to label pathways for their suggested relevance in *M. m.* T2D.

## 3. Results

**Figure 2:**
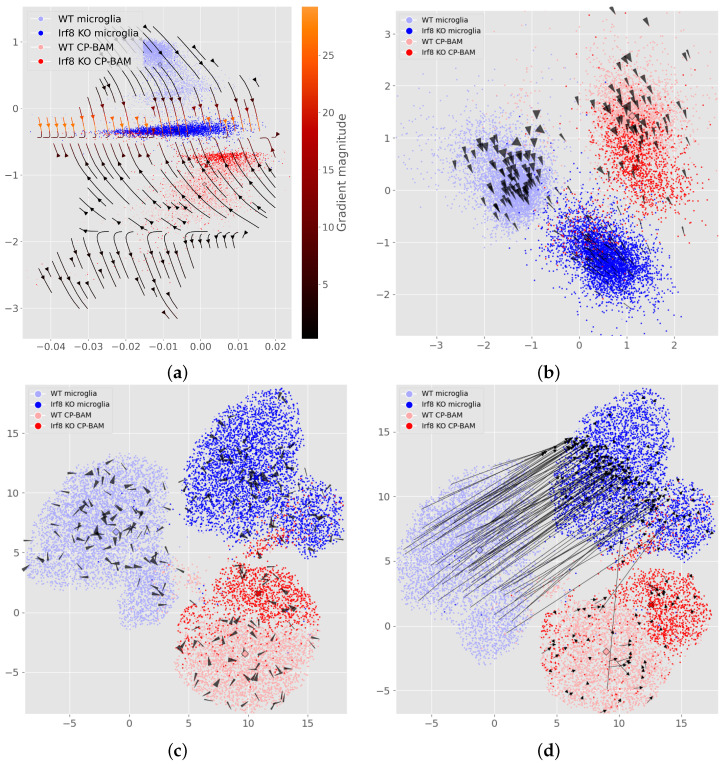
Perturbation flow maps showing directions of *decreasing Irf8* on the mouse brain dataset of Van Hove et al. [10]. Arrows illustrate directions of the negative *mean* gradient of a small subset of six genes inferred to be co-regulated with *Irf8*. That is, the flows on this figure describe the path of gradual knockdown. Cell types are microglia and choroid-plexus border-associated macrophages (CP-BAM), both brain-resident macrophages that regulate inflammation and clear debris. (**a**) Streamplot of gradients on a 2-dimensional latent space; (**b**) PCA of 32-dimensional latent space; (**c**) UMAP of 32-dimensional latent space; (**d**) UMAP with arrows from 400 gradient steps with stepsize δ=−0.001.

**Predicting knockout response.** To evaluate the utility of the perturbation flows, a case study on the *Irf8*-cKO dataset [10] is first performed. Visualizing the negative gradient of *Irf8*-expression in latent space shows the effect of gradual knockdown, moving the wild type population to the knockout population, both for microglia and CP-BAM (Figure 2a), and more evidently for a higher-dimensional latent space (Figure 2b). Similarly, effects of gene overexpression are successfully simulated (see Appendix E, Figure A2).

**Figure 3:**
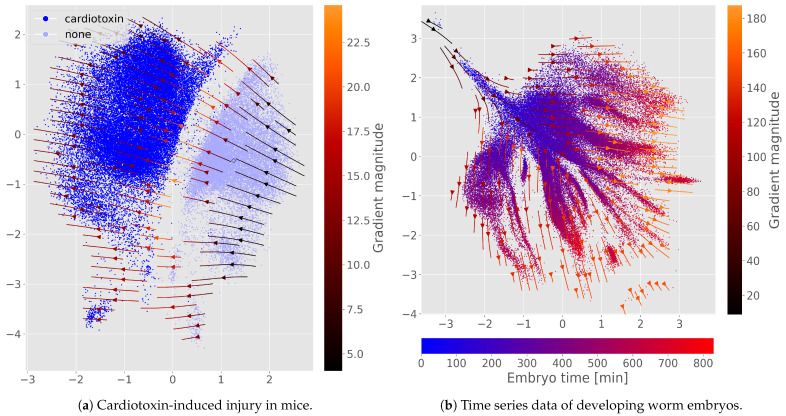
Perturbation flow maps when using auxiliary output variables. Arrows are a streamplot visualization of the gradients. Gradients are computed from the output of cardiotoxin classification or embryo time regression; the flows describe the path of gradually *increasing* the variable of interest, i.e., probability of cardiotoxin injury or embryo age.

**Predicting injury response.** Next, we consider the dataset of cardiotoxin-induced mouse injury [11]. A binary variable is added to the output features to indicate cardiotoxin injury. This output variable is included in the objective function with a scaled binary cross-entropy loss term, $\alpha L_{\mathrm{CTX}}$. Training with just 10% of injury labels, the model still achieves 99.0% accuracy in predicting the cardiotoxin label on the held-out test set. Visualizing the gradient of the cardiotoxin prediction in latent space (Figure 3a) shows how toxin affects the latent samples, simulating changes in their expression profiles. As expected, perturbation vectors strictly point from wild type to experimentally perturbed samples.

**Predicting temporal dynamics.** Dimensionality reduction on the *C. e.* embryogenesis dataset was found to distinctly subcluster cell types according to the age of the embryo sample [12]. Adding this embryo time as a continuous output feature enables including an additional L1 loss term $\alpha L_{\mathrm{time}}$ in our objective function. Training again with just 10% of available time labels, Figure 3b shows that the gradient of time predictions can be used to infer how cells develop, and is well aligned with the observed sample times. The latent space further stays subdivided in distinct cell types (illustrated by Appendix E, Figure A3).

**Figure 4:**
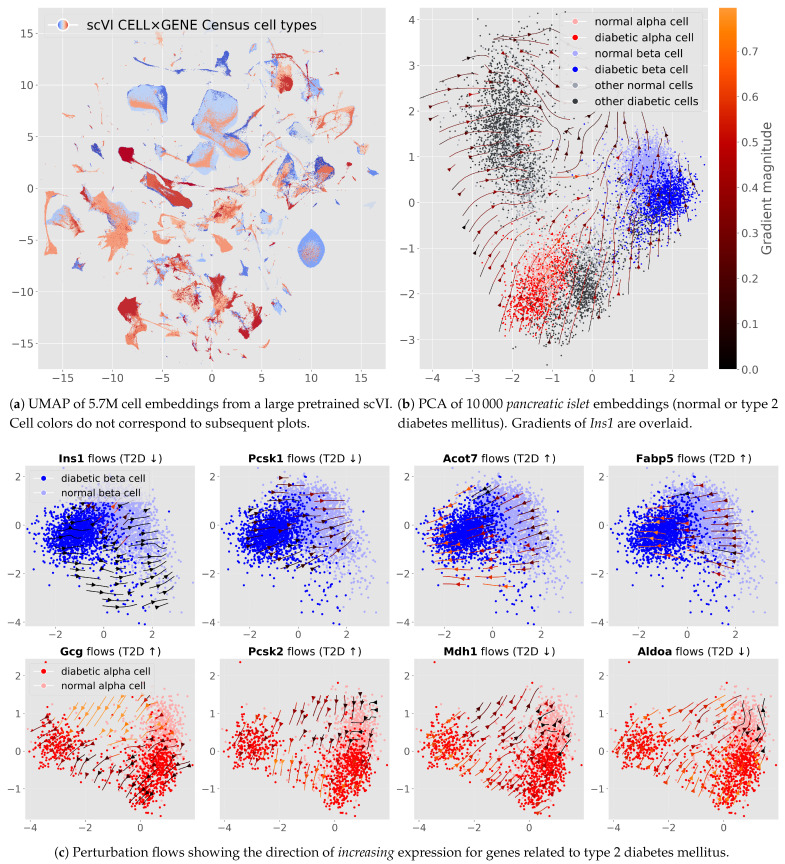
Top row: (**a**) UMAP projection of all embeddings from the scVI Census model and (**b**) PCA projection of islet cells with *Ins1* gradients. Bottom rows (**c**): simulated in silico perturbations showing directions for β- and α-related gene interventions (*increasing* expression); up- and down-arrows in titles indicate whether ground-truth disease expression is usually considered up- or down-regulated.

**Predicting influences of genes related to a complex disease.** Similarly to gene under- and overexpression, we perform genetic perturbations on pancreatic cells to explore drivers of T2D. We use a pretrained scVI model based on the CELL×GENE Discover Census; this model embeds more than 5.7 M *M. m.* cells in a 50-dimensional latent space (Figure 4a). We randomly sample 10,000 cells belonging to either the *normal* or *type 2 diabetes mellitus* populations from the islet of Langerhans tissue (Figure 4b). We do not introduce any new data, and the model was never trained on any perturbation labels. However, analyzing genetic perturbation flows reveals how various model genes related to type 2 diabetes *do indeed* create flows from healthy normal cells to pathological diabetic cells (Figure 4c). The perturbation flows particularly affect β- and α-cells, which are specialized cells that secrete insulin and glucagon, respectively; their dysfunction is central to type 2 diabetes [26,27].

In particular, we observe that increasing *Ins1* in β-cells corresponds to gradients from diabetic → normal (Figure 4c, top-left), consistent with its role in lowering blood glucose. Increasing *Gcg* in α-cells aligns with a normal → diabetic shift (Figure 4c, bottom-left), reflecting its opposing endocrine role. We observe a similar pattern with *Pcsk1* in β-cells and *Pcsk2* in α-cells, respectively (*Pcsk1* enhances proinsulin → insulin conversion; *Pcsk2* promotes proglucagon → glucagon processing). Next, we focus on genes involved in metabolic pathways (Figure 4c, right-most four panels). Here, increasing *Acot7* and *Fabp5* in β-cells correlates with a normal → diabetic shift, consistent with their known overexpression in diabetes. In α-cells, higher *Mdh1* and *Aldoa* correctly correlate with a diabetic → normal shift.

**Figure 5:**
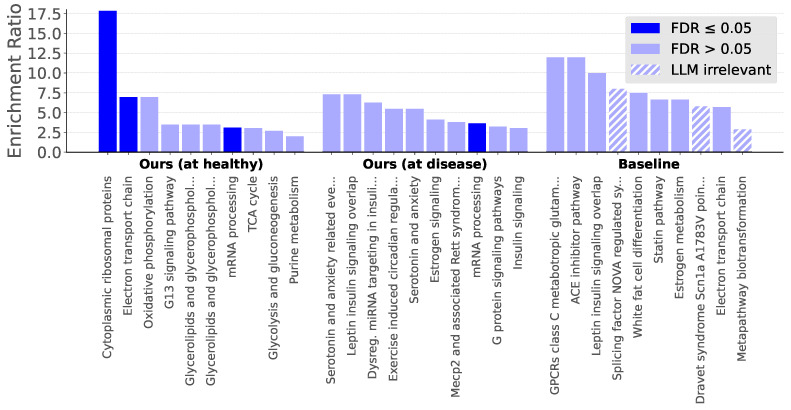
Results from overrepresentation analysis based on WikiPathways. Pathways are labeled by their false discovery rate (FDR) and LLM-inferred relevance. As a baseline, we decode the NB means at the median latent of each condition to obtain per-gene $m^{\left(1\right)}$ and $m^{\left(0\right)}$, and score genes by the symmetric change $b_{i}=\frac{m^{\left(1\right)}-m^{\left(0\right)}}{\frac{1}{2}\left(m^{\left(1\right)}+m^{\left(0\right)}\right)}$. Genes with top 200 largest magnitudes are used for analysis.

By scoring how well a genetic perturbation is aligned with an experimentally observed perturbation, we inferred top genes related to T2D in mice. Figure 5 shows pathways from WikiPathways that are overrepresented in the top 200 scoring genes. While an ordinary baseline did not identify any pathways at the false discovery rate ≤0.05, extending our method for enrichment managed to locate multiple significant pathways. Further, through our mechanistic analyses with LLM AI agents, we distinguish that enriched pathways are more relevant for T2D in *M. m.* than pathways from the baseline. Appendix D shows a sample of the agentic pathway analyses.

## 4. Discussion

### 4.1. Single-Cell Models Encode Perturbation Effects Without Using Labels

Generative decoders can be queried to infer the effects of perturbations on gene expression—even without perturbation labels. This is evidenced by the perturbation flow maps of Figure 2, Figure 3 and Figure 4, demonstrating an intuitive and visual interpretation of these perturbation effects. For each dataset, the generative model converged with low reconstruction error (Appendix B).

**Figure A2:**
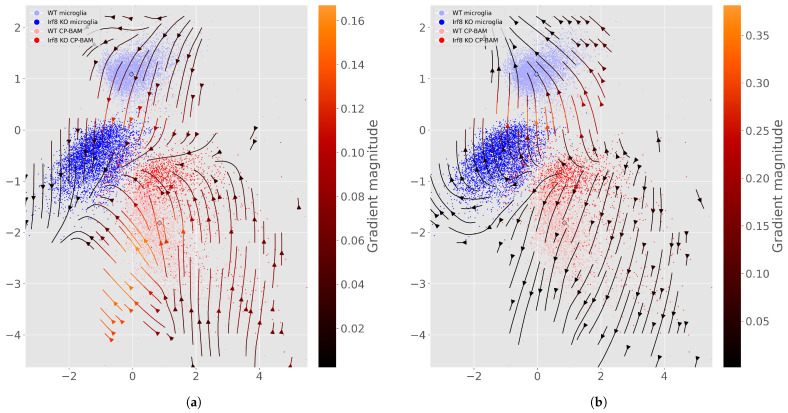
Perturbation flow maps for gene perturbations on the *Irf8*-cKO dataset. The gradient is aggregated as the mean over genes which are differentially under- or overexpressed when comparing wild type and cKO populations. (**a**) Mean negative gradient of differentially underexpressed genes; (**b**) Mean negative gradient of differentially overexpressed genes.

**Figure A3:**
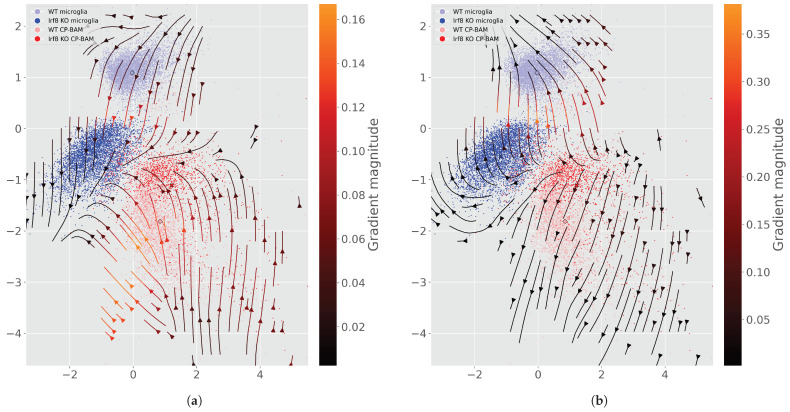
Latent space representations for the *C. e.* embryogenesis dataset. Latent variables are shown with two different labeling schemes: embryo measurement time and cell type. This illustrates how the model captures continuous development while maintaining distinct cellular populations. (**a**) Re-print of Figure 3b for easier comparison; (**b**) Cell type annotations for the *C. e.* latent space.

First, we find that knockdown flows recover known biology. The decoder’s knockdown predictions for the *Irf8*-cKO dataset align with findings by Van Hove et al. [10] emphasizing *Irf8*’s significance in microglia. Flows point from WT samples to cKO samples when decreasing expression of *Irf8* (Figure 2a,b). Conversely, flows approximately reverse when considering the mean gradient of genes, which are differentially overexpressed in the cKO set (Appendix E, Figure A2).

### 4.2. Auxiliary Outputs Extend to Treatment and Time

The perturbation concept is easily generalized as demonstrated in the cases of cardiotoxin injury and embryonic development. In these contexts, gradients highlight different cellular dynamics—e.g., transitions from control to cardiotoxin-altered states and temporal patterns during development. Even small amounts of labeled data can be used to achieve a general understanding of the whole dataset—and thus simulate perturbation trajectories for new unlabeled cells. Differential expression analysis *along* these trajectories could help inform target discovery and drug design.

### 4.3. Flow Maps Scale to High-Dimensional Latents and Can Improve Projections

While intuitive in two dimensions, higher-dimensional latent spaces capture richer effects. Figure 2b shows how a larger dimensionality could better encode the effect of *Irf8* for CP-BAM cells. Similarly, Figure 4 uses an off-the-shelf pretrained model; these typically have latent spaces of 50 dimensions. Inferred perturbations can also aid the visualization of relationships *across clusters* in UMAP-reduced spaces (Figure 2d), suggesting a route to recover global structure typically lost in UMAP.

### 4.4. Type 2 Diabetes: Probing a Pretrained Model at Scale

We probe a publicly released scVI decoder trained on the CELL×GENE Discover Census (∼5.7 M mouse cells) without any finetuning or perturbation supervision. From these data, we sample 10,000 islets of Langerhans cells and visualize gradients as vector fields after PCA projection. Figure 4a shows the global embedding; Figure 4b shows pancreatic islets of either *normal* or *type 2 diabetes mellitus* status. This disease *primarily* separates from normal cells in β- and α-cell regions—these are the principal endocrine populations that secrete insulin and glucagon, respectively. We overlaid gradients of the insulin output (*Ins1*) in this PCA, and notice how an *increase* in insulin relates to disease → healthy.

In Figure 4c, we zoom in on β- and α-cells respectively with PCAs fitted on each cell type. Here, gene-specific gradient fields for increasing expression align with known disease axes: genes downregulated in T2D for β-cells (*Ins1*, *Pcsk1*, *Mdh1*, *Aldoa*) point from diabetic to normal β-cell regions, whereas genes upregulated in T2D for α-cells (*Pcsk2*, *Gcg*) and stress/metabolic markers (*Acot7*, *Fabp5*) point from normal toward diabetic regions. The fields are thus locally meaningful to the appropriate cell types. Our results clearly indicate that large pretrained decoders already encode gradients aligned with complex-disease axes and can be used directly for hypothesis generation.

### 4.5. Output Features Can Be Scored According to an Observed Perturbation

The alignment score in Equation (3) provides a local, label-free readout of how each decoder output aligns with an empirical perturbation axis. The evaluation set Z controls locality: restricting to specific cell types or neighborhoods yields cell-state-conditioned rankings, while averaging across broader regions yields global summaries. This provides users with a large degree of control.

After scoring genes based on their alignment with a healthy-to-disease axis in T2D, our enrichment analysis found a roster of pathways relevant to the disease. Compared to a baseline enrichment approach, our identified pathways were *more significant* and *more relevant* based on an LLM-powered large-scale assessment (Figure 5).

Extensions are straightforward. One may weigh by gradient magnitude to couple *direction* and *local effect size*, ${\tilde{s}}_{i}={\mathrm{avg}}_{z\in Z}\left(\nabla y_{i}{\left(z\right)}^{⊤}a/{∥a∥}_{2}\right)$, or integrate gradients along the perturbation axis. To this end, comparing locally sampled gradients against an optimal transport map could drastically improve scores—albeit at a higher computational cost. Finally, scores transfer to any decoder output, enabling unified ranking of genes, treatments, and even sets of variables. Including electronic health records or other modalities could provide additional utility. This setting would also benefit from our method’s ability to compute scores for local groups—and even individuals. Overall, alignment-based scoring turns pretrained decoders into compact hypothesis engines for target nomination and pathway discovery.

### 4.6. Summary and Directions

We introduced a model-agnostic gradient probe that turns any single-cell decoder into a simulator of perturbations. Across genetic, chemical, and temporal settings, the resulting flow fields recovered known transitions and supported hypothesis generation. *Zero-shot* probing (i.e., without any finetuning) of an scVI model trained on 5.7 M cells correctly revealed diabetes-aligned flows in islet β- and α-cells. Using gradients to score genes led to powerful feature enrichment that can be evaluated in local, controlled areas of latent space. This enrichment was seen to identify pathways that highly correlate with the underlying disease condition. Future work may further investigate this empirical direction, look into incorporating gradient magnitudes, or further improve perturbation alignment scoring. In brief, our approach requires only a decoder, optional lightweight auxiliary heads, and scales to high-dimensional latents of pretrained general-use models. Our results indicate that modern generative models already encode vast disease- and perturbation-relevant information that can be accessed by simple automatic differentiation.

## Figures and Tables

**Figure A1:**
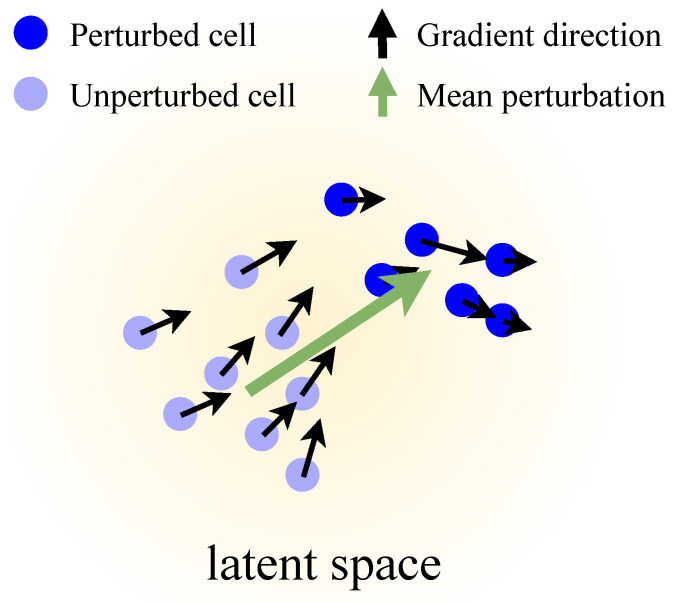
An alignment score for each gene is computed by measuring the average cosine similarity between sampled gradients and an observed perturbation vector.

## Data Availability

All datasets analyzed in this study are openly available from the original publications cited in the manuscript [10,11,12,13]. Code for reproducing the analyses is available at the repository https://github.com/yhsure/perturbations (accessed on 9 November 2025). No new datasets were generated.

## Abbreviations

The following abbreviations are used in this manuscript:

cKO	conditional knockout
*C. e.*	*Caenorhabditis elegans*
CP-BAM	choroid-plexus border-associated macrophages
FDR	false discovery rate
HVG	highly variable genes
KL	Kullback–Leibler divergence
LLM	large language model
*M. m.*	*Mus musculus*
NB	negative binomial
PCA	principal component analysis
scRNA-seq	single-cell RNA sequencing
scVI	single-cell variational inference (model name)
T2D	type 2 diabetes mellitus
UMAP	uniform manifold approximation and projection
UMI	unique molecular identifier
VAE	variational autoencoder
WT	wild type

## Appendix A. On Using a Pretrained scVI Model

For pretrained scVI models, we access the decoder through the underlying PyTorch modules rather than the high-level wrappers. This exposes the generative call and the decoder distribution px, which will allow computing gradients with respect to latents *z* through torch.autograd.
**Rationale.** Our perturbation flows require $\nabla_{z}y_{i}\left(z\right)$, where $y_{i}$ is the decoder output for gene *i* (or an auxiliary head). The usual scVI model API makes this impossible as it detaches tensors and hides intermediate objects. However, calling the internal m.module.generative method for a model m keeps the computation graph intact. We utilize this function to make a minimal decoder forward pass.

The px object from the output provides mean expression via get_normalized(“scale”) (NB rate on a normalized scale). This is suitable for defining $y_{i}\left(z\right)$ and taking gradients. In the following pseudocode, the jac function is similar to jacobian from torch.autograd.functional—taking a function and its inputs to return the Jacobian—although we use a faster implementation.

### Minimal Decode and Gradient Functions (Pseudocode)

def decode(z, library, batch_idx):

   out := m.module.generative(z=z, library=library, batch_index=batch_idx)

   px  := out[‘‘px’’]

   exp := px.get_normalized(‘‘scale’’)

   return exp

def grad_wrt_i(z, i, library, batch_idx):

   J := jac(lambda x, l, b: decode(x, l, b)[:, i],

         z, library, batch_idx)

   return J

This is the only necessary code to use pretrained models for our perturbation flows. Some model instances may require other inputs than *z*, library sizes and batch indices.

## Appendix B. Tabular Overview of Trained Models

**Table A1:** Tabular overview of the trained models (results across 5 runs). Root mean squared error (RMSE) and mean absolute error (MAE) compare log-transformed model outputs to log-transformed mean-scaled counts. *Task* denotes either decimal accuracy (cardiotoxin prediction) or mean L1 norm (embryogenesis regression). Adjusted rand index (ARI) relies on k-means clustering and is computed for timepoint (cardiotoxin data) and cell type for other datasets.

	Train Set				Test Set			
Instance	ARI	RMSE	MAE	Task	ARI	RMSE	MAE	Task
*Irf8* cKO	0.72 ± 0.02	0.19 ± 0.00	0.23 ± 0.00	−	0.74 ± 0.02	0.19 ± 0.00	0.23 ± 0.00	−
32D *Irf8* cKO	0.87 ± 0.02	0.16 ± 0.00	0.21 ± 0.00	−	0.71 ± 0.17	0.17 ± 0.00	0.21 ± 0.00	−
Cardiotoxin	0.77 ± 0.05	0.27 ± 0.00	0.31 ± 0.00	1.00 ± 0.00	0.76 ± 0.05	0.27 ± 0.00	0.31 ± 0.00	0.99 ± 0.00
Embryogenesis	0.33 ± 0.04	0.31 ± 0.01	0.25 ± 0.00	11.37 ± 3.81	0.33 ± 0.04	0.31 ± 0.01	0.26 ± 0.00	32.04 ± 1.75

## Appendix D. Pathway Relevance According to LLM AI Agents

**Table A2:** Pathway relevance to type 2 diabetes *according to LLM AI agents*. “Verdict” indicates the final *Prompt 2* verdict, and in parentheses, the count of Yes votes from *Prompt 1*. All entries, including resources, are written word-for-word by the LLM agent. This table is a sample; for transparency, all results are displayed on https://github.com/yhsure/perturbations/blob/main/data/llm_pathway_responses.json (all links accessed on 7 November 2025).

Pathway	Verdict	Explanation	Relevant Resources
**Cytoplasmic ribosomal proteins**	Yes (3/3)	There is experimental and transcriptomic evidence in mouse and human islets linking cytoplasmic (and mitochondrial) ribosomal proteins and ribosome biogenesis to β-cell protein synthesis, mitochondrial dysfunction, impaired insulin secretion and dysregulated insulin/AKT signaling — mechanisms directly relevant to T2D pathogenesis in *Mus musculus*.	Ribosomal biogenesis regulator DIMT1 controls β-cell protein synthesis, mitochondrial function, and insulin secretion (2022), https://pubmed.ncbi.nlm.nih.gov/35148993/. Mitoribosome insufficiency in β cells is associated with type 2 diabetes-like islet failure (2022), https://pubmed.ncbi.nlm.nih.gov/35804190/. Ribosomal Protein Mutations Induce Autophagy through S6 Kinase Inhibition of the Insulin Pathway (2014), https://www.ncbi.nlm.nih.gov/pmc/articles/PMC4038485/.
**Dravet syndrome Scn1a A1783V point mutation model**	No (0/3)	The Scn1a A1783V (Nav1.1) Dravet mouse is a CNS-focused loss-of-function epilepsy model with published phenotypes confined to brain/behavior, seizures and respiratory dysfunction; there is no evidence this SCN1A α-subunit variant perturbs pancreatic β-cell function or produces insulin resistance/T2D in mouse. Voltage-gated Na^+^ channel isoforms relevant to islet excitation–secretion are *Scn3a*/*Scn9a* (α subunits) and the β subunit *Scn1b*, not *Scn1a*, and loss/variation in those genes — not *SCN1A* A1783V — has been linked to altered insulin/glucagon secretion.	Na^+^ current properties in islet α- and β-cells reflect cell-specific *Scn3a* and *Scn9a* expression (2014), https://pubmed.ncbi.nlm.nih.gov/25172946/. Sodium channel β1 regulatory subunit deficiency reduces pancreatic islet glucose-stimulated insulin and glucagon secretion (2008), https://pmc.ncbi.nlm.nih.gov/articles/PMC2654754/. Dravet variant *SCN1A* A1783V impairs interneuron firing predominantly by altered channel activation (2021), https://pubmed.ncbi.nlm.nih.gov/34776868/. Proteomic signature of the Dravet syndrome in the genetic *Scn1a*-A1783V mouse model (2021), https://pubmed.ncbi.nlm.nih.gov/34144125/.
**mRNA processing**	Yes (3/3)	Strong experimental and genetic evidence in mice (and conserved mammalian mechanisms) shows mRNA processing — especially alternative splicing, RNA-binding proteins and m6A RNA modification — directly regulates insulin production, β-cell function and insulin signalling, and perturbations produce glucose-homeostasis defects and diabetes-like phenotypes in *Mus musculus*.	N6-adenosine methylation controls the translation of insulin mRNA (2023), https://pmc.ncbi.nlm.nih.gov/articles/PMC11756593/. Haplo-Insufficiency of the Insulin Receptor in the presence of a splice-site mutation in *Ppp2r2a* results in a novel digenic mouse model of type 2 diabetes (2018), https://pmc.ncbi.nlm.nih.gov/articles/PMC5947768/. mRNA Processing: An Emerging Frontier in the Regulation of Pancreatic β Cell Function (2020), https://pmc.ncbi.nlm.nih.gov/articles/PMC7490333/.
**Serotonin and anxiety**	Yes (3/3)	Strong, mechanistic mouse data show serotonergic signaling directly regulates pancreatic β-cell function (TPH1, *Htr3a*/*Htr2b*) and central 5-HT receptors (*Htr2c* in POMC neurons) control glucose homeostasis; anxiety/stress-related alterations in 5-HT circuits in mouse models further modulate glycemia and insulin sensitivity, supporting high relevance of the serotonin–anxiety axis to T2D in *Mus musculus*.	Serotonin Regulates Adult β-Cell Mass by Stimulating Perinatal β-Cell Proliferation (2019), https://pmc.ncbi.nlm.nih.gov/articles/PMC6971487/. Functional role of serotonin in insulin secretion in a diet-induced insulin-resistant state (2015), https://pubmed.ncbi.nlm.nih.gov/25426873/. Serotonin 2C receptors in pro-opiomelanocortin neurons regulate energy and glucose homeostasis (2013), https://www.jci.org/articles/view/70338.
**Estrogen signaling**	Yes (3/3)	Strong experimental evidence in *Mus musculus* shows estrogen signaling (primarily via ERα, also ERβ/GPER) modulates hepatic and muscle insulin sensitivity, suppresses hepatic gluconeogenesis, preserves β-cell lipid homeostasis/function and prevents diet- or ovariectomy-induced insulin resistance — mechanisms directly relevant to T2D pathogenesis in mice.	Estrogen Improves Insulin Sensitivity and Suppresses Gluconeogenesis via the Transcription Factor Foxo1 (2018), https://pubmed.ncbi.nlm.nih.gov/30487265/. Estrogen signaling prevents diet-induced hepatic insulin resistance in male mice with obesity (2014), https://pubmed.ncbi.nlm.nih.gov/24691030/. Estrogen receptor activation reduces lipid synthesis in pancreatic islets and prevents β cell failure in rodent models of type 2 diabetes (2011), https://pubmed.ncbi.nlm.nih.gov/21747171/. Estrogen signaling pathway — *Mus musculus* (KEGG mmu04915), https://www.kegg.jp/pathway/mmu04915.
**Metapathway biotransformation**	No (1/3)	Metapathway biotransformation (phase I/II xenobiotic metabolism) is a hepatic/cellular detoxification module that is reproducibly altered in mouse models of obesity/T2D and can modulate insulin sensitivity (e.g., CYP epoxygenases/EETs), but it is not a core insulin-signalling or glucose-homeostasis pathway driving T2D in *Mus musculus*. Thus it is indirectly relevant and may modify disease severity, but it is not “highly” relevant as a primary T2D pathway.	Metapathway biotransformation (WP1251) — *Mus musculus* (2024), https://www.wikipathways.org/pathways/WP1251.html. Cytochrome P450 epoxygenase-derived epoxyeicosatrienoic acids contribute to insulin sensitivity in mice and in humans (2017), https://pubmed.ncbi.nlm.nih.gov/28352940/. CYP2J2 attenuates metabolic dysfunction in diabetic mice by reducing hepatic inflammation via the PPARδ (2015), https://pmc.ncbi.nlm.nih.gov/articles/PMC4329496/.
**Exercise-induced circadian regulation**	Yes (3/3)	Strong experimental evidence in *Mus musculus* shows (1) timed exercise entrains peripheral clocks in muscle and liver and modifies CLOCK/BMAL1/PER2 and SIRT1–NAD^+^ pathways, (2) chrono-exercise alters insulin sensitivity, GLUT4-mediated glucose uptake and mitochondrial quality in diabetic mouse models, and (3) circadian disruption causes glucose intolerance and insulin resistance in mice—together supporting high relevance of exercise-induced circadian regulation for T2D in mouse.	Chrono-Aerobic Exercise Optimizes Metabolic State in DB/DB Mice through CLOCK–Mitophagy–Apoptosis (2022), https://pubmed.ncbi.nlm.nih.gov/36012573/. Aerobic exercise timing affects mitochondrial dynamics and insulin resistance by regulating the circadian clock protein expression and NAD^+^-SIRT1-PPARα-MFN2 pathway in the skeletal muscle of high-fat-diet-induced diabetes mice (2024), https://pubmed.ncbi.nlm.nih.gov/39715985/. Circadian Disruption across Lifespan Impairs Glucose Homeostasis and Insulin Sensitivity in Adult Mice (2023), https://pubmed.ncbi.nlm.nih.gov/38393018/. Skeletal muscle insulin sensitivity shows circadian rhythmicity which is independent of exercise training status (2018), https://www.ncbi.nlm.nih.gov/pmc/articles/PMC6121032/. Sleep, circadian rhythms, and type 2 diabetes mellitus (2021), https://www.ncbi.nlm.nih.gov/pmc/articles/PMC8939263/.
